# Retraction Note: Propofol suppresses growth, migration and invasion of A549 cells by down-regulation of miR-372

**DOI:** 10.1186/s12885-024-12247-0

**Published:** 2024-04-15

**Authors:** Hai Sun, Dengyu Gao

**Affiliations:** Department of Anesthesiology, China-Japan Union Hospital of Jilin University, No.126, Xiantai Street, 130033 Changchun, Jilin China

**Retraction Note: BMC Cancer 18, 1252 (2018)**

10.1186/s12885-018-5175-y

The Editors have retracted this article following an investigation report by the Ministry of Science and Technology of the People’s Republic of China. Their investigation found attempts to tamper with the data. The Editors therefore no longer have confidence in the integrity of the data in this article.

Author, Hai Sun has not responded to any correspondence from the editor/publisher about this retraction. The publisher has not been able to obtain a current email address for author, Dengyu Gao.

